# Supplementary material for: IER5 Promotes Ovarian Cancer Cell Proliferation and Peritoneal Dissemination
Source: Cancers (Basel). 2025 Feb 11;17(4):610. doi: 10.3390/cancers17040610 (PMC11853144; doi:10.3390/cancers17040610)
Supplement: Supplementary file 1 [file cancers-17-00610-s001.zip › cancers-3327713-supplementary.pdf]

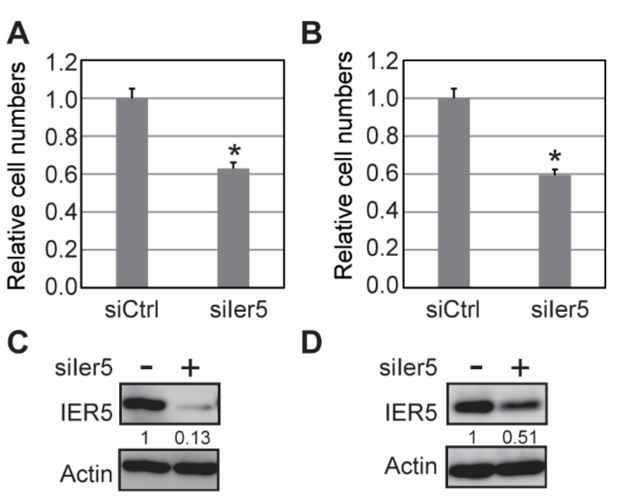


**Figure S1**: *Ier5* knockdown reduces cell numbers. (A,B) Relative number of ID8G (A) and ID8G−Om2 (B) cells 72 h post−siRNA (*Ier5*) transfection. (C,D) Protein expression of IER5 in HM−1 (C) and MOV (D) cells treated with siIer5. Error bars represent mean ± SD (n = 3) and the '*p*' values were calculated using '*t*' test method. * $p < 0.05$. Relative densities of protein bands (except for actin) are numbered below the bands in the western blots.
